# A Supramolecular Nanoassembly of Lenvatinib and a Green Light-Activatable NO Releaser for Combined Chemo-Phototherapy

**DOI:** 10.3390/pharmaceutics15010096

**Published:** 2022-12-28

**Authors:** Francesca Laneri, Nadia Licciardello, Yota Suzuki, Adriana C. E. Graziano, Federica Sodano, Aurore Fraix, Salvatore Sortino

**Affiliations:** 1 PhotoChemLab, Department of Drug and Health Sciences, University of Catania, 95125 Catania, Italy; 2 Department of Materials and Life Sciences, Sophia University, Tokyo 102-8554, Japan; 3 Department of Pharmacy, University of Napoli Federico II, 80131 Napoli, Italy

**Keywords:** light, nitric oxide, chemotherapeutic, cyclodextrin polymers, combination therapy

## Abstract

The chemotherapeutic Lenvatinib (LVB) and a nitric oxide (NO) photodonor based on a rhodamine antenna (RD-NO) activatable by the highly compatible green light are supramolecularly assembled by a β-cyclodextrin branched polymer (PolyCD). The poorly water-soluble LVB and RD-NO solubilize very well within the polymeric host leading to a ternary supramolecular nanoassembly with a diameter of ~55 nm. The efficiency of the NO photorelease and the typical red fluorescence of RD-NO significantly enhance within the polymer due to its active role in the photochemical and photophysical deactivation pathways. The co-presence of LVB within the same host does not affect either the nature or the efficiency of the photoinduced processes of RD-NO. Besides, irradiation of RD-NO does not lead to the decomposition of LVB, ruling out any intermolecular photoinduced process between the two guests despite sharing the same host. Ad-hoc devised Förster Resonance Energy Transfer experiments demonstrate this to be the result of the not close proximity of the two guests, which are confined in different compartments of the same polymeric host. The supramolecular complex is stable in a culture medium, and its biological activity has been evaluated against HEP-G2 hepatocarcinoma cell lines in the dark and under irradiation with visible green light, using LVB at a concentration well below the IC_50_. Comparative experiments performed using the polymeric host encapsulating the individual LVB and RD-NO components under the same experimental conditions show that the moderate cell mortality induced by the ternary complex in the dark increases significantly upon irradiation with visible green light, more likely as the result of synergism between the NO photogenerated and the chemotherapeutic.

## 1. Introduction

Lenvatinib (LVB) is a multikinase inhibitor (MKI) exerting anti-malignant effects by inhibiting many tyrosine kinase receptors such as vascular endothelial growth factor (VEGF) and fibroblast growth factor receptors [1,2,3,4]. LVB has proven to be more efficacious than sorafenib in unresectable hepatocellular carcinoma (HCC) [5,6]. However, the poor aqueous solubility under physiological conditions [7], the low bioavailability [8], and the serious side effects, including hypertension, nausea, vomiting, weight loss, edema, and rash [9], represent some of the main limitations of this drug. Furthermore, analogously to many chemotherapeutics, multidrug resistance (MDR) remains a major hurdle for its long-term anti-cancer activity [10]. Therefore, the development of new approaches is very challenging for both improving the therapeutic efficacy of LVB and reducing its side effects.

The combination of chemotherapy with light-triggered treatment modalities as a suitable strategy has been receiving increasing attention, especially over the last decade. Among light-activated therapeutic approaches, photodynamic therapy (PDT) is the most known and finds application in clinics [11]. It mainly bases on the cytotoxic action of singlet oxygen (^1^O_2_) produced catalytically by a collisional energy transfer process between the excited triplet state of suitable photosensitizers (PS) and the nearby molecular oxygen [12]. Prototype examples of PDT combined with MKI, such as sorafenib and LVB, have been recently reported [13,14]. In the frame of light-activated therapeutic modalities, nitric oxide (NO) based PDT, namely NO-PDT, has come to the limelight over the last few years [15] to be used either in alternative or in combination with traditional PDT [16]. In fact, besides its well-known role in the bioregulation of physiological processes [17,18], NO radical exerts a remarkable anti-cancer action [19]. This can be the result of a direct cytotoxic effect of NO [17,18] or an indirect effect due to the inhibition by NO of the efflux pumps mainly responsible for MDR [20,21]. The anti-cancer activity of NO is, however, strictly dose-dependent [22,23,24,25]. For such a reason, NO-PDT exploits suitable NO photodonors (NOPDs) whose NO release can be accurately controlled in space and time with great accuracy by tuning the intensity and location of the excitation light. Analogously to ^1^O_2_, NO has a short lifetime (~5 s in tissue), absence of charge, good lipophilicity, short diffusion radius (<200 µm), multitarget reactivity towards proteins, lipids, and DNA, and lack of MDR. However, in contrast to ^1^O_2_ production, NO generation is not dependent on the presence of oxygen, representing a great advantage under hypoxic conditions where traditional PDT fails [26].

NO-PDT combination with conventional chemotherapeutics has been reported [27,28,29,30,31], and only in a recent work the key role of NO combined with MKI has been proposed as a suitable approach to improve cancer treatments with this class of drugs [32]. 

**Scheme 1:**
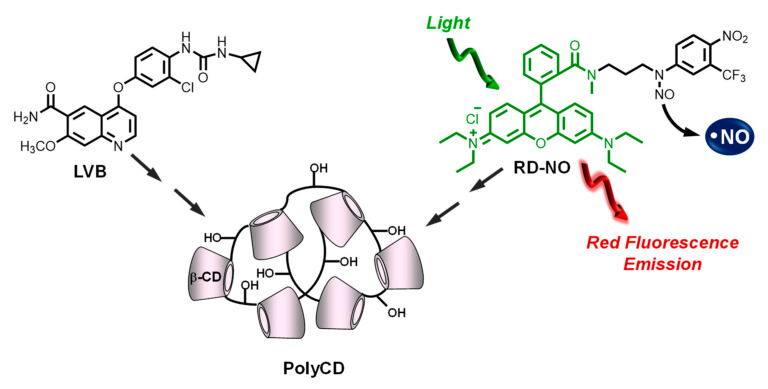
Molecular structures of the guests LVB and RD-NO, which are supramolecularly assembled in the branched polymeric host PolyCD.

We recently reported the first combination of MKI sorafenib with NO-PDT [33]. It has been achieved by encapsulating the chemotherapeutic within a β-cyclodextrin (β-CD) branched polymer covalently integrating an NOPD activatable with blue light. This construct showed a remarkable enhancement of the anti-cancer activity at a concentration of the drug below the IC_50_ due to a remarkable synergistic effect with the photogenerated NO [33]. These encouraging results prompted us to extend our approach to LVB and to improve it by using the NOPD based on a rhodamine antenna, RD-NO (Scheme 1), which is activatable with the much more biocompatible green light and emits in the red region [34]. In this paper, we report the preparation, characterization, and biological evaluation of a novel supramolecular ternary complex in which LVB and RD-NO are both supramolecularly co-encapsulated within the β-CD branched polymer PolyCD, used as suitable carrier system (Scheme 1).

## 2. Materials and Methods

### 2.1. Materials

LVB mesylate was purchased from MedChemExpress (HY-10981, Madrid, Spain). All other chemicals were purchased by Sigma-Aldrich and used as received. PolyCD was prepared by cross-linking β-CD with epichlorohydrin, under strongly alkaline conditions, following a described method [35]. A mixture of different molecular weight compounds was obtained in the polycondensation reaction. Separation was performed with size exclusion chromatography (SEC) with water-compatible high-performance columns using pullulan standards. The β-CD content in PolyCD was ~70% *w*/*w*, as determined based on NMR spectra. RD-NO and NBF were synthesized according to our previously reported procedures [34,36]. The concentration of LVB was determined by UV-Vis spectroscopy, using a molar extinction coefficient of 72.350 M^–1^ cm^–1^ in MeOH at λ = 241 nm and 62.100 M^–1^ cm^–1^ at λ = 245 nm when complexed within PolyCD. All solvents used for the spectrophotometric studies were at spectrophotometric grade. MilliQ water was used for the solubilization of the polymer and for the chemical and photochemical experiments.

### 2.2. Instrumentation

UV-Vis absorption and fluorescence emission spectra were recorded with a JascoV-560 spectrophotometer and a Spex Fluorolog-2 (mod. F-111) spectrofluorimeter, respectively, in air-equilibrated solutions, using either quartz cells with a path length of 0.1 cm or 1 cm. Fluorescence lifetimes were recorded with the same fluorimeter equipped with a TCSPC Triple Illuminator. The samples were irradiated by pulsed diode excitation sources (LED) at 455 nm, and the decays were monitored at 590 nm. The system allowed the measurement of fluorescence lifetimes with a time resolution of 200 ps. The multiexponential fit of the fluorescence decay was obtained using the following equation:

I(t) = Σα_i_exp(−t/τi)

Absorption spectral changes were monitored by irradiating the sample in a thermostated quartz cell (1 cm path length, 3 mL capacity) under gentle stirring, using a continuum laser with λ_exc_ = 532 nm, ~100 mW and having a beam diameter of ~1.5 mm.

Direct monitoring of NO release in solution was performed by amperometric detection (World Precision Instruments, Sarasota, Fl, USA), with an ISO-NO meter equipped with a data acquisition system, and based on direct amperometric detection of NO with short response time (<5 s) and sensitivity range 1 nM–20 µM. The analog signal was digitalized with a four-channel recording system and transferred to a PC. The sensor was accurately calibrated by mixing standard solutions of NaNO_2_ with 0.1 M H_2_SO_4_ and 0.1 M KI according to the reaction:

4H^+^ + 2I^−^ + 2NO_2_^−^ → 2H_2_O + 2NO + I_2_

Irradiation was performed in a thermostated quartz cell (1 cm path length, 3 mL capacity) using the continuum laser with λ_exc_ = 532 nm. NO measurements were carried out under stirring with the electrode positioned outside the light path in order to avoid NO signal artifacts due to photoelectric interference on the ISO-NO electrode.

The size distribution was determined on dynamic light scattering Horiba LS 550 apparatus equipped with a diode laser with a wavelength of 650 nm.

### 2.3. NO Photorelease and Fluorescence Quantum Yields

NO photorelease quantum yield, Φ_NO_, was determined at λ_exc_ = 532 nm within the 20% transformation of RD-NO by using the following equation:

Φ_NO_ = [tr-RD-NO] × V/t × (1–10^−A^) × I

where [tr-RD-NO] is the concentration of phototransformed RD-NO, V is the volume of the sample, t is the irradiation time, A is the absorbance of the sample at the excitation wavelength, and I the intensity of the excitation light source. The concentration of the photo-transformed RD-NO was determined by UV-Vis absorption, monitoring the absorption changes at λ = 380 nm and using a Δε_380_ = 7100 M^–1^ cm^–1^. I was calculated by potassium ferrioxalate actinometry. 

The value of fluorescence quantum yield, Φ_f_, was determined using optically matched solutions at the excitation wavelength of RD-NO within the PolyCD and Rhodamine B in EtOH as a standard (Φ_f_ = 0.79) [37] and calculated through the following equation:

Φ_f_ = Φ_f_(s) (In^2^/I(s)n^2^(s)) (1)

where Φ_f_(s) is the fluorescence quantum yield of the standard; I and I(s) are the areas of the fluorescence spectra of RD-NO and the standard, respectively; n and n(s) is the refraction index of the solvents used for compound and standard. Absorbance at the excitation wavelength was less than 0.1 in all cases.

### 2.4. Preparation of the Supramolecular Complexes

A stock solution of LVB, RD-NO, or both components in MeOH was utilized, and the solvent was evaporated under reduced pressure at 25 °C to form a thin film. The resulting film was rehydrated with 3 mL of phosphate buffer solution (PBS) 10^−2^ M, pH 7.4 of PolyCD (2 mg mL^−1^) by stirring for 5 h at room temperature to obtain the binary complexes, PolyCD/LVB and PolyCD/RD-NO, and the ternary complex PolyCD/LVB/RD-NO. For the biological assays and some of the chemical evaluations, the complexes were prepared by rehydrating the films with a solution of PolyCD (2 mg mL^−1^) in DMEM high glucose (without phenol red), as detailed in the biological experiment section.

### 2.5. Biological Experiments

#### 2.5.1. Cell Lines

Hepatocelular carcinoma cell line (HepG2) was from American Type Culture Collection (ATCC, Manassas, VA, USA). Cells were cultured in Dulbecco’s modified Eagle’s medium with high-glucose and without phenol-red (DMEM, Sigma-Aldrich, Milan, Italy) containing 10% of fetal bovine serum (FBS), L-glutamine, 100 U ml^−1^ of penicillin and 100 μg ml^−1^ of streptomycin (all from Sigma-Aldrich, Milan, Italy) in a humidified incubator (Heracell 150, Thermo Scientific, Waltham, MA, USA) at 37 °C, 95% rel. humidity and 5% CO_2_. 

#### 2.5.2. Determination of IC_50_

For the determination of the IC_50_, a stock solution of LVB (10 mM) was prepared in DMSO, and it was diluted in an FBS-free medium in order to have scalar dilutions for in vitro experiments (0.06; 0.13; 0.78; 1.56; 3.125; 6.25; 12.5; 25; 50; 100 µM). Control culture received an equal amount of vehicles (DMSO). The incubation was carried out for 24 or 4 h. The short-term incubation of 4 h was followed by an additional 24 h of cell growth in a drug-free medium with serum (4 h +24 h) and then exposed to 3-[4,5-dimethylthiazol-2-yl]-2,5 diphenyl tetrazolium bromide (MTT) salt for the determination of cell viability.

#### 2.5.3. Cell Viability

Cells were detached from the bottom of flasks by trypsin-EDTA solution (Sigma-Aldrich, Milan, Italy), counted in a Bürker chamber, and plated into 96-well plates (EuroClone, Milan, Italy) at a density of 1 × 10^4^ for 48 h until the monolayer reached a confluency of approximately 80%. For in vitro evaluation, PolyCD was dissolved in an FBS-free culture medium (2 mg mL^−1^). LVB was loaded into the PolyCD by dissolving the thin film obtained after drying a methanol solution with a solution of PolyCD (2 mg mL^−1^ in FBS-free culture medium) under 5-h magnetic stirring in the dark, reaching a final LVB concentration of 50 μM. To obtain PolyCD loaded only with RD-NO or with both LVB and RD-NO, films of RD-NO were dissolved in the proper volume of PolyCD alone or PolyCD loaded with LVB in order to obtain a final concentration of RD-NO of 6 μM. Cells were exposed to PolyCD alone or in combination with LVB in the presence and absence of RD-NO for 4 h prior to irradiation with light. Cells were then washed, and the phenol red-free and FBS-free medium were added. The experimental plates were divided into two experimental groups: one was maintained in the dark, while the other was exposed to a 150 W Xe lamp (the measured power at 30 cm away from the lamp where the samples were placed was ~50–60 mW) through a 500 nm cut-off filter for 10, 20, or 30 min. In each group, untreated cells were maintained as a control. After the irradiation, the FBS-free medium was removed, and the complete medium was added. All the plates were placed back into the incubator. After 24 h, the cellular metabolic activity was assessed by MTT assay. Briefly, after the established time, the medium was replaced with 180 μL of medium and 20 μL of MTT solution (stock solution at 5 mg mL^−1^), and the wells were left incubated for 3 h at 37 °C. Under this condition, mitochondrial dehydrogenases of viable cells conversed the tetrazolium ring of MTT into formazan crystals that were solubilized in 100 μL of DMSO. The absorbance was measured at λ = 570 nm in a microplate reader (AMR-100, Allsheng, Zhejian, China). The cytotoxicity index was calculated using the control cells as 100% viability, and the IC_50_ was extrapolated from the dose-response graph by nonlinear regression analysis using GraphPad Prism Software version 7.00 (GraphPad Software, La Jolla California USA). Cell viability after light exposure was calculated as percentage ± SD with respect to untreated cells kept in the dark.

#### 2.5.4. Fluorescence Microscopy

To analyze the uptake and the distribution of PolyCD/RD-NO without and with LVB, cells were plated on sterilized glass coverslips inserted into 24-well culture plates and incubated for 4 h with the prepared systems. Then, cells were washed with Dulbecco’s Phosphate Buffered Saline (DPBS, Lonza, Walkersville, MD, USA) and fixed by a 20 min-incubation with formaldehyde (4% in DPBS, Sigma-Aldrich, Milan, Italy). The cover slips were mounted on microscope slides (Epredia, Braunschweig, Germany) by using the UltraCruz Hard-set mounting medium with 4′,6-diamidino-2-phenylindole (DAPI) to counterstain the nuclei (Santa Cruz Biotechnology, Dallas, TX, USA). Samples were observed using a Zeiss Axioplan microscope (Carl Zeiss, Milan, Italy) equipped with a 100× objective, epifluorescence filters (DAPI: Excitation G365, Beamsplitter FT 395, Emission LP 420; red fluorescence: Excitation BP 510-560, Beamsplitter FT 580, Emission LP 590), and an Axicam camera (Carl Zeiss, Milan, Italy). 

## 3. Results and Discussion

CD branched polymers represent an intriguing class of scaffolds for the supramolecular assembling of multiple guests since they offer the possibility of interactions with diverse binding sites, such as the 3D macromolecular network and the hydrophobic CD cavities [38,39,40,41]. This feature not only enhances the solubility of hydrophobic guests but also avoids their self-association propensity [38,39,40,41]. This aspect is crucial in the case of photoresponsive compounds since it may preclude any response to light due to self-quenching phenomena. Furthermore, the modular character of supramolecular assembling does not require any additional synthetic efforts, permits the easy regulation of the relative concentrations of multiple guests, and facilitates the delivery process in a biological environment [42]. 

On these bases, we chose the branched polymer PolyCD as a suitable host and carrier system for LVB and RD-NO. This polymer consists of β-CD units interconnected by epichlorohydrin spacers to form glyceryl cross-linked β-CD polymer. It is highly soluble in a water medium, well-tolerated in vivo, and able to entrap a variety of guests with enhanced stability constants and payloads when compared with the unmodified β-CD. Moreover, it has been extensively used in our group to integrate a number of phototherapeutics and effectively deliver them into cancer cells [43,44,45,46]. 

**Figure 1:**
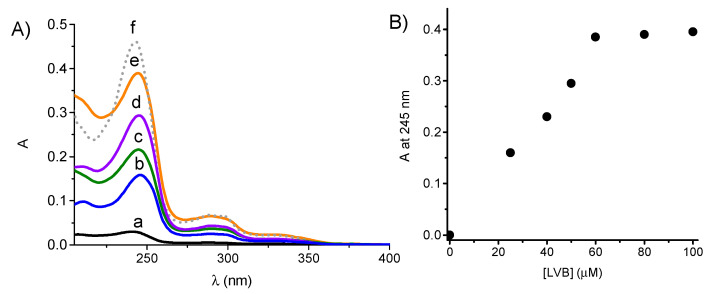
(**A**) Absorption spectrum of LVB in PBS (pH 7.4) at 4.1 µM (a) and in the presence of PolyCD (2 mg mL^−1^) at 25 µM (b), 40 µM (c), 50 µM (d) and 60 µM I (e). The spectrum of a 60 µM LVB solution in methanol is shown for the sake of comparison (f). (**B**) Absorbance value at 245 nm of LVB at different concentrations in the presence of PolyCD. T = 25 °C; cell path = 0.1 cm.

LVB is poorly soluble in PBS solution with a solubility limit of ~4.1 µM (~1.7 μg mL^−1^). Solubility studies were performed by using a concentration of PolyCD of 2 mg mL^−1^ (see experimental). Figure 1 shows a significant enhancement of the drug solubility in the presence of the polymer, reaching a limiting value of ~60 µM (~25.5 μg mL^−1^), which is more than one order of magnitude than that observed in the absence of the polymer. Interestingly, apart from a slight red shift of the absorption maxima due to the localization of the drug in an environment of different polarity, the absorption profile of the encapsulated LVB is very similar to that observed in the methanol solution (f in Figure 1A). This finding rules out any intra- aggregation of the drug, which is instead present mainly in its monomeric form. The complex is stable for several days under ambient conditions, as demonstrated by the unchanged position and absorbance values of the absorption spectrum.

**Figure 2:**
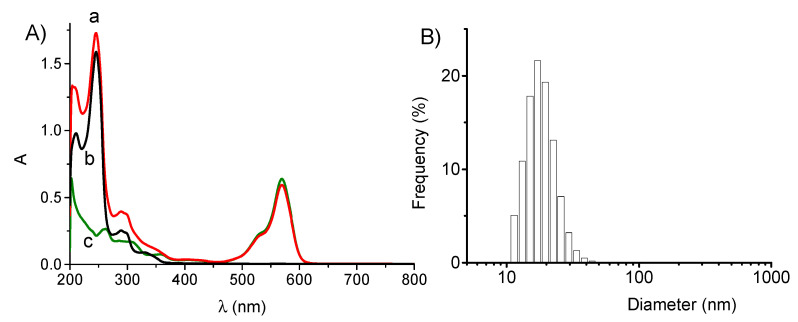
(**A**) Absorption spectra of PolyCD (2 mg mL^−1^) in PBS (pH 7.4) co-loaded with LVB (25 µM) and RD-NO (6 µM) (a) and, for the sake of comparison, loaded only with LVB (b) and RD-NO (c). T = 25 °C; cell path = 1 cm. (**B**) Hydrodynamic diameter of sample a in Figure 2A.

In the frame of our interest in photoactivatable NO releasers, we have recently developed the compound RD-NO (see Scheme 1) in which a rhodamine antenna is covalently linked to an N-nitroso appendage [34]. Excitation of the rhodamine chromophore with the highly biocompatible green light triggers the NO release through an intramolecular electron transfer without precluding the typical red fluorescence emission of rhodamine, which is useful for imaging [34]. This compound is not soluble in a water medium but is soluble in the presence of the PolyCD polymer both without and with LVB. Figure 2A shows the absorption spectra of the polymeric host loaded with both RD-NO and LVB and, for comparison, those of the polymer loaded with the single guest components at the same concentration. The spectrum of the ternary complex (a in Figure 2A) matches very well the spectral profile obtained by summing the spectra of the binary complexes obtained by loading the polymeric host with the single components (b and c in Figure 2A). This accounts for the absence of relevant interactions between RD-NO and LVB in the ground state. Furthermore, the unchanged position of all the absorption maxima indicates that the presence of RD-NO does not induce any rearrangement of LVB within the polymer and vice versa. Dynamic light scattering measurements indicate an average hydrodynamic diameter for the supramolecular assembly of about 50 nm in the presence of both guests (Figure 2B), a value in excellent agreement with those already observed for the same polymer co-encapsulating different guests [43,44,45,46].

**Figure 3:**
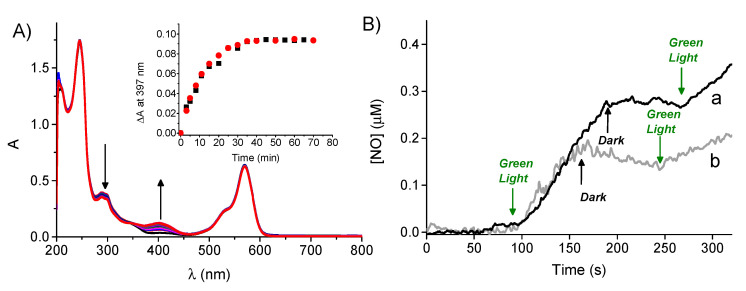
(**A**) Absorption spectral changes observed upon 532 nm light irradiation of a PBS (pH 7.4) solution of PolyCD (2 mg mL^−1^) co-loaded with LVB (25 µM) and RD-NO (6 µM). The inset shows the absorbance changes at 397 nm of PolyCD (2 mg mL^−1^**)** co-loaded with LVB (25 µM) and RD-NO (6 µM) (λ) and, for comparison, the same host in the absence of LVB (ν). (**B**) NO release profile observed for PolyCD (2 mg mL^−1^) co-loaded with LVB (25 µM) and RD-NO (6 µM) (a) and, for comparison, the same host in the absence of LVB (b). T = 25 °C.

As shown in Figure 2, LVB absorption falls in the UV spectral region and does not interfere with the typical absorption band of RD-NO in the visible region centered at 570 nm. This spectral condition permits the selective excitation of RD-NO with the highly biocompatible green light. Figure 3A shows the spectral absorption changes observed upon irradiation of the PolyCD/LVB/RD-NO ternary complex at 532 nm. They show the formation of a new absorption at 397 nm and bleaching at 291 nm, accompanied by the presence of a clear isosbestic point at 348 nm, indicative of a clean photochemical reaction. This photochemical profile is very similar to that previously observed for the free RD-NO in methanol/water [34] and is in agreement with the light-triggered release of NO. Note that both the efficiency and nature of the photochemical process are not influenced by the co-presence of LVB, as demonstrated by the identical absorbance changes observed for the RD-NO-loaded PolyCD in the absence of LVB (see inset Figure 3A) and the lack of any spectral modification in the correspondence of the absorption band of LVB at 245 nm.

NO photorelease from the ternary and, for comparison, from the binary supramolecular assemblies was demonstrated by the direct detection of NO. Figure 3B shows the NO release profile observed through amperometric detection, which clearly indicates that NO is produced under green light excitation, stops in the dark, and restarts as the light is turned on again. According to the identical photochemical kinetics (inset Figure 3A), the NO photorelease efficiency was not affected by the presence of LVB. Quantitative analysis of the photolysis process revealed that the polymeric nanocarrier has a profound effect on the quantum yield of NO generation. A value of Φ_NO_ = 4 ± 0.5 × 10^−3^ was, in fact, observed under these conditions, which is ~4-fold larger than that observed for the free RD-NO [34]. This larger photoreactivity is not uncommon for radical-mediated photodecomposition occurring within polymer environments. In our case, it may be the result of the active role the polymeric network plays in providing easily abstractable hydrogens close to the anilinyl radical intermediate involved in the mechanism of the NO photorelease. This has already been reported for a similar nitroso-derivative NOPD encapsulated in polymeric hosts [36].

**Figure 4:**
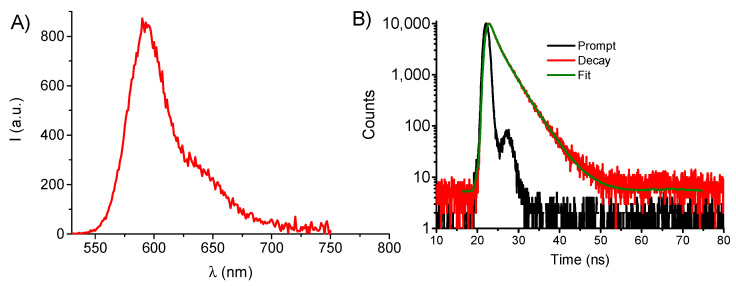
(**A**) Fluorescence emission spectrum observed at λ_exc_ = 520 nm light excitation for a PBS (pH 7.4) solution of PolyCD (2 mg mL^−1^) co-loaded with LVB (25 µM) and RD-NO (6 µM). (**B**) Fluorescence decay and the related fitting of the same solution recorded at λ_exc_ = 455 nm and λ_em_ = 590 nm. T = 25 °C.

RD-NO combines NO photorelease regulated by green light to remarkable fluorescence emission, which is useful for cell tracking by fluorescence imaging. Figure 4A shows the typical fluorescence emission spectrum of RD-NO observed after its encapsulation within PolyCD in the presence of LVB. The fluorescence quantum yield was Φ_f_ = 0.40, which is the same as that observed in the absence of LVB but almost two-fold larger than that observed for the free guest RD-NO (Φ_f_ = 0.22) [34]. This indicates a reduction of the non-radiative decay pathways, probably due to a restriction of the free rotation motion of the fluorophore embedded within the polymeric network. Accordingly, the fluorescence dynamic Figure 4B) showed a bi-exponential decay with a shorter lifetime τ_1_ = 1.4 ns, the same as what was observed for the free guest [34], and a longer component with τ_1_ = 3.6 ns, with the relative weight of ~40% and 60%, respectively. 

**Figure 5:**
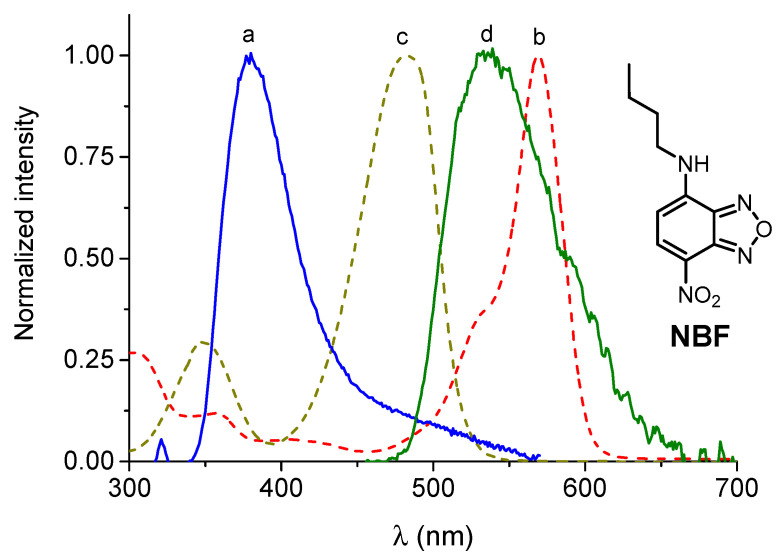
Normalized fluorescence spectra of LVB (a) (λ_exc_ = 240 nm), NBF (d) (λ_exc_ = 440 nm), and absorption spectra of NBF (c) and RD-NO (b). All guests are loaded in PolyCD (2 mg mL^−1^) dissolved in PBS (pH 7.4). T = 25 °C.

The preservation of the photochemical properties of a photoactivatable component, once co-encapsulated with another guest in the same host, is not a trivial result and is a fundamental requisite for combining photo- with chemo-therapeutic effects. Potential photoinduced energy/electron transfer between the two guests in close proximity can indeed lead to undesired processes, which preclude the final goal. In our case, the photochemical and photophysical behaviors of RD-NO are even improved after its encapsulation in the host nanocarrier and are not affected by the co-presence of LVB. This finding rules out any quenching by the chemodrug on the excited states of RD-NO, accounting for not close proximity of the two guests, which are probably confined in different locations of the polymeric host. To gain more insight into this important aspect, we carried out ad-hoc devised Förster Resonance Energy Transfer (FRET) experiments, which permit to shed light on the spatial proximity of two guests. LVB shows a fluorescence emission at ~380 nm (a in Figure 5). However, this emission does not show a significant overlap with the absorption spectrum of RD-NO (b in Figure 5), which is a necessary condition for the occurrence of FRET between these two components. Accordingly, we did not observe any FRET upon excitation of LVB in the presence of RD-NO. This spectral limitation, thus, precludes obtaining information into the relative location of the guests in the host. To overcome this limitation, we used the nitrobenzofurazan derivative NBF (Figure 5) as a suitable additional guest mediator. This compound is highly hydrophobic, dissolves well within the PolyCD, and exhibits spectral properties that make it an ideal energy acceptor and energy donor towards LVB and RD-NO, respectively. In fact, LVB emission significantly overlaps the NBF absorption (see spectra a and c in Figure 5), and the NBF fluorescence emission significantly overlaps with the RD-NO absorption (see spectra d and b in Figure 5).

**Figure 6:**
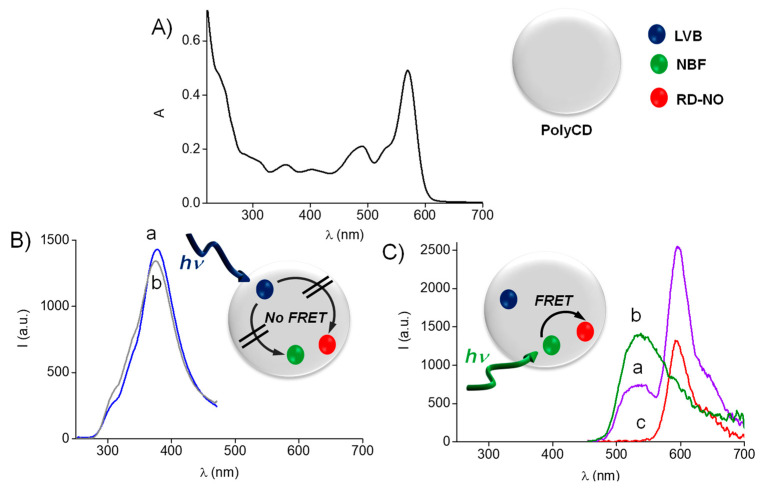
(**A**) Absorption spectrum of PolyCD (2 mg mL^−1^) in PBS (pH 7.4) co-loaded with LVB, RD-NO, and NBF (5 µM). (**B**) Fluorescence emission spectra of the sample as in (**A**) (a) and PolyCD (2 mg mL^−1^) in PBS (pH 7.4) loaded only with LVB at the same concentration (b) recorded at λ_exc_ = 240 nm. (**C**) Fluorescence emission spectra of the sample as in (**A**) (a) and PolyCD (2 mg mL^−1^) in PBS (pH 7.4) loaded only with NBF (b) and RD-NO (c) recorded at λ_exc_ = 445 nm.

LVB, NBF, and RD-NO (all at the same concentration) can be simultaneously co-loaded in the PolyCD (2 mg mL^−1^), as demonstrated by the absorption spectrum showing the typical spectral features of all guests (Figure 6A). This reflects well the sum of the absorption spectra of every single component, ruling out any significant interaction between them in the ground state. In the first experiment, we recorded the fluorescence emission spectrum of the sample loaded with all three guests by exciting LVB (potential energy donor) at 240 nm, and we compared it with that of a similar sample in the absence of NBF (potential energy acceptor). As shown in Figure 6B, no quenching of LVB emission was observed, suggesting the absence of FRET between LVB and NBF and thus accounting for their not close proximity in the host. In a second experiment, we recorded the fluorescence emission spectrum of the sample loaded with all three guests by exciting NBF (potential energy donor) at 445 nm (a in Figure 6C), and we compared it with that of similar samples loaded with either NBF (potential energy donor) alone (b in Figure 6C) or RD-NO (potential energy acceptor) alone (c in Figure 6C). The results obtained show a significant quenching of the NBF fluorescence in the presence of RD-NO accompanied by a corresponding increase in the RD-NO emission, in accordance with an effective FRET process between these two guests and accounting for their close proximity. Therefore, taken together, all these results will confirm that the therapeutic components LVB and RD-NO are confined in different compartments of the polymeric host and very distant from each other. This feature allows the photochemical and photophysical properties of RD-NO within the nanocarrier to be preserved even in the presence of LVB, which, in turn, is not decomposed upon light excitation of RD-NO (i.e., by undesired photoinduced bimolecular processes), thus preserving its chemotherapeutic action. Note that the FRET experiments reported in Figure 6C were well reproduced in the presence of a culture medium (5% in serum) for several days, confirming the stability of the supramolecular complex even under these experimental conditions.

**Figure 7:**
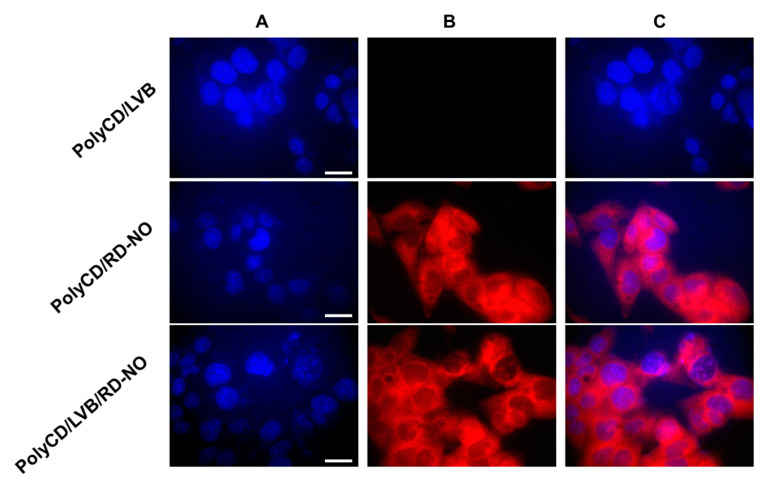
Fluorescence microscopy analysis of HEP-G2 hepatocarcinoma cell lines treated with PolyCD (2 mg mL^−1^) in PBS (pH 7.4) loaded with LVB (25 µM), RD-NO (6 µM) and both components and stained with DAPI. The cells were analyzed with a DAPI emission filter (**A**), a rhodamine emission filter (**B**), or by merging images (**A**,**B**) (**C**). Scale bar = 50 µM.

The excellent emissive properties of RD-NO in the host nanocarrier and their preservation, even after co-encapsulation with LVB, allowed for exploring the cellular uptake of the supramolecular assembly by fluorescence microscopy. Figure 7 shows representative fluorescence images of HEP-G2 hepatocarcinoma cell lines obtained after 4 h incubation with PolyCD loaded with RD-NO, LVB, or both components. It is evident that in all cases, the typical red fluorescence originating from RD-NO accounts for a localization mainly at the cytoplasmatic level, and it is not affected by the presence of LVB. 

**Figure 8:**
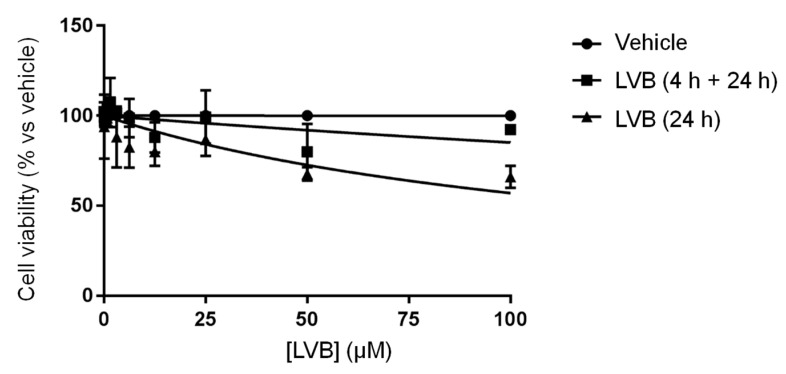
Viability of HEP-G2 hepatocarcinoma cells as a function of the concentration of free LVB.

Cell mortality experiments were carried out with the above-mentioned cell lines. In a preliminary experiment, we determined the IC_50_ value of the chemodrug by investigating the cell viability as a function of the free LVB concentration in a serum-free medium. Under our experimental condition, the 4 h incubation with LVB did not alter cell viability, and 24 h treatment had a limited role in inducing mitochondrial dysfunctions. According to these results, the IC_50_ was 132.6 ± 26.8 µM at 24 h and 572.1 ± 235.6 µM at 4 h + 24 h (Figure 8).

**Figure 9:**
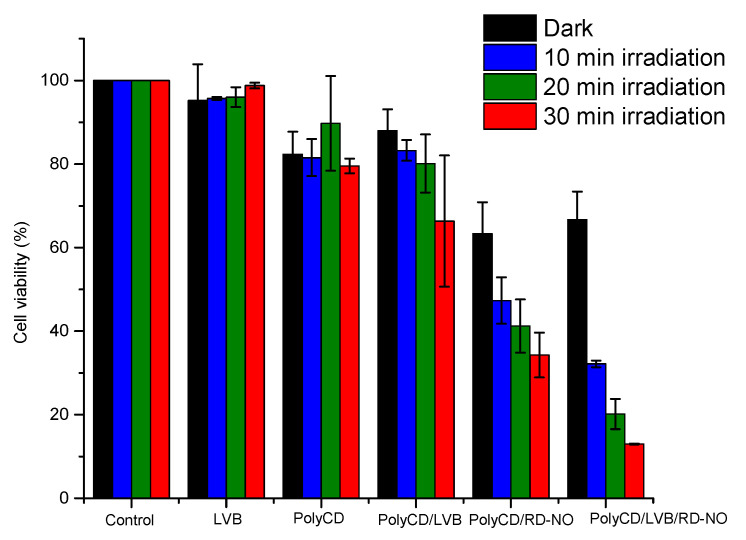
Cell viability was observed 24 h after incubating HEP-G2 hepatocarcinoma cells with free LVB, PolyCD, and the supramolecular complexes PolyCD/LVB, PolyCD/RD-NO, and PolyCD/LVB/RD-NO in the dark and upon different irradiation times (occurred after the first 4 h of incubation) at λ_exc_ > 500 nm. [LVB] = 50 µM; [RD-NO] = 6 µM; [PolyCD] = 2 mg mL^−1^.

The biological activity of the ternary supramolecular assembly was tested under visible light excitation and in the dark and, for the sake of comparison, was compared to that of the binary complexes containing the individual either LVB or RD-NO under identical experimental conditions. These experiments were performed with LVB at a concentration of 50 µM, which is well below the IC_50_, and RD-NO at a concentration of 6 µM with irradiation not longer than 30 min, which allows generating NO only at moderately cytotoxic doses. The overall results are illustrated in Figure 9.

The PolyCD polymeric host is well tolerated both in the dark and under light irradiation. Besides, under the chosen experimental conditions, LVB-only loaded PolyCD showed negligible toxicity in the dark and a moderate reduction in cell viability only with longer irradiation time. In contrast, PolyCD loaded with the photoactive RD-NO induced levels of toxicity depending on the irradiation time according to the production of moderate NO doses. Interestingly, a significant reduction in cell viability was observed in the case of the ternary complex containing both LVB and RD-NO. The potentiation indexes (% viability free LVB/% viability polyCD/LVB/RD-NO) were ~3, 5, and 8 at 10, 20, and 30 min of irradiation, respectively. It should be emphasized that since the presence of LVB in the supramolecular assembly does not affect the NO photorelease efficiency (see Figure 3B), the enhancement of cell mortality cannot be due to higher NO concentration photogenerated by the complex. Therefore, the occurrence of a notable additive/synergistic action between the chemotherapeutic and the NO appears clear. A possible explanation for this synergism can be related to recent studies that have demonstrated that MKI, including LVB, induces the generation of reactive oxygen species (ROS) in human HCC cell lines in vivo and in vitro [47,48]. In our case, the formation of ROS, such as superoxide anion, may lead to a diffusion-controlled reaction with NO, generating the highly cytotoxic peroxynitrite [49]. Investigations addressed to gain insights into the mechanisms at the basis of such synergistic action definitely deserve attention. 

## 4. Conclusions

Our results have demonstrated that the anti-cancer activity of LVB enhances by its combination with an NO photoreleaser through a supramolecular complex based on a biocompatible polymeric host nanocarrier. This latter (i) increases the solubility of LVB by more than one order of magnitude with respect to that observed for the free drug in water, (ii) enhances the photochemical and photophysical performances of the NO photoreleaser and (iii) represents an ideal container, which permits the confinement of the two guests in different compartments, avoiding any intermolecular communication between them and, thus, preserving their individual properties. 

The synergistic action between LVB used at a concentration well below its IC_50_ and the NO generated at moderated cytotoxic doses through the appropriate light tuning validate the combination of photoregulated NO with MKI as a suitable approach to enhance their anti-cancer action. Compared with our previous work on sorafenib, the present work represents a step forward in terms of biocompatibility of excitation light (from blue to green) to activate the NO delivery, with a gain of more than 100 nm towards lower energy. Furthermore, the modular character of the supramolecular assembling of both therapeutic components does not require any additional synthetic efforts, permitting the easy regulation of the relative concentrations of the two guests and facilitating the delivery process in a biological environment. 

Finally, the use of spontaneous NO releasers in combination with MKI has also been recently proposed as a suitable strategy to exploit the role of NO as a vasodilator able to reduce hypertension [50,51], which is one of the main side effects of this class of chemotherapeutics [9]. In this view, and considering the well-known critical dichotomous biological effects of NO strictly depending on its concentration [23,24,25,26,27], our findings may also open up interesting avenues in the perspective of in vivo applications not only to enhance the anti-cancer activity of MKI but also to reduce one of their major side effects.

## References

[1] Schlumberger M., Tahara M., Wirth L.J., Robinson B., Brose M.S., Elisei R., Habra M.A., Newbold K., Shah M.H., Hoff A.O. (2015). Lenvatinib versus placebo in radioiodine-refractory thyroid cancer. N. Engl. J. Med..

[2] Kiyota N., Schlumberger M., Muro K., Ando Y., Takahashi S., Kawai Y., Wirth L., Robinson B., Sherman S., Suzuki T. (2015). Subgroup analysis of Japanese patients in a phase 3 study of lenvatinib in radioiodine-refractory differentiated thyroid cancer. Cancer Sci..

[3] Al-Salama Z.T., Syed Y.Y., Scott L.J. (2019). Lenvatinib: A Review in Hepatocellular Carcinoma. Drugs.

[4] Cabanillas M.E., Habra M.A. (2016). Lenvatinib: Role in thyroid cancer and other solid tumors. Cancer Treat. Rev..

[5] Kudo M., Finn R.S., Qin S., Han K.H., Ikeda K., Piscaglia F., Baron A., Park J.W., Han G., Jassem J. (2018). Levantinib versus sorafenib in first-line treatment of patients with unresectable hepatocellular carcinoma: A randomised phase 3 non-inferiority trial. Lancet.

[6] Forner A., Reig M., Bruix J. (2018). Hepatocellular carcinoma. Lancet.

[7] Hong M., Li S., Ji W., Qi M.H., Ren G.B. (2021). Cocrystals of Lenvatinib with Sulfamerazine and Salicylic Acid: Crystal Structure, Equilibrium Solubility, Stability Study, and Anti- Hepatoma Activity. Cryst. Growth Des..

[8] Chen S., Cao Q., Wen W., Wang H. (2019). Targeted therapy for hepatocellular carcinoma: Challenges and opportunities. Cancer Lett..

[9] Lenvatinib in Combination with Everolimus. https://www.fda.gov/drugs/resources-information-approved-drugs/lenvatinib-combination-everolimus.

[10] Housman G., Byler S., Heerboth S., Lapinska K., Longacre M., Snyder N., Sarkar S. (2014). Drug Resistance in Cancer: An Overview. Cancers.

[11] Agostinis P., Berg K., Cengel K.A., Foster T.H., Girotti A.W., Gollnick S.O., Hahn S.M., Hamblin M.R., Juzeniene A., Kessel D. (2011). Photodynamic therapy of cancer: An update. CA Cancer J. Clin..

[12] Celli J.P., Spring B.Q., Rizvi I., Evans C.L., Samkoe K.S., Verma S., Pogue B.W., Hasan T. (2010). Imaging and photodynamic therapy: Mechanisms, monitoring, and optimization. Chem. Rev..

[13] Guo T., Lin W., Chen W., Huang Y., Zhu L., Pan X. (2020). Photodynamic Therapy in Combination with Sorafenib for Enhanced Immunotherapy of Lung Cancer. J. Biomed. Nanotechnol..

[14] Zong J., Peng H., Qing X., Fan Z., Xu W., Du X., Shi R., Zhang Y. (2021). pH-Responsive Pluronic F127-Lenvatinib-Encapsulated Halogenated Boron-Dipyrromethene Nanoparticles for Combined Photodynamic Therapy and Chemotherapy of Liver Cancer. ACS Omega.

[15] Ostrowski A.D., Ford P.C. (2009). Metal complexes as photochemical nitric oxide precursors: Potential applications in the treatment of tumors. Dalton Trans..

[16] Fraix A., Sortino S. (2018). Combination of PDT photosensitizers with NO photodononors. Photochem. Photobiol. Sci..

[17] Ignarro L.J., Freeman B.A. (2017). Nitric Oxide: Biology and Pathobiology.

[18] Ignarro L.J. (2009). Special Journal Issue on Nitric Oxide Chemistry and Biology. Arch. Pharm. Res..

[19] Wink D.A., Mitchell J.B. (1998). Chemical biology of nitric oxide: Insights into regulatory, cytotoxic, and cytoprotective mechanisms of nitric oxide. Free Radic. Biol. Med..

[20] Sinha B.K., Bortner C.D., Mason R.P., Cannon R.E. (2018). Nitric oxide reverses drug resistance by inhibiting aptase activity of P-glycoprotein in human multidrug-resustant cancer cells. Biochim. Biophys. Acta Gen Subj..

[21] Riganti C., Miraglia E., Viarisio D., Costamagna C., Pescarmona G., Ghigo D., Bosia A. (2005). Nitric oxide reverts the resistance to doxorubicin in human colon cancer cells by inhibiting the drug efflux. Cancer Res..

[22] Fukumura D., Kashiwagi S., Jain R.K. (2006). The role of nitric oxide in tumour progression. Nat. Rev. Cancer.

[23] Chang C.F., Diers A.R., Hogg N. (2015). Cancer cell metabolism and the modulating effects of nitric oxide. Free Radic. Biol. Med..

[24] Moncada S., Erusalimsky J.D. (2002). Does nitric oxide modulate mitochondrial energy generation and apoptosis?. Nat. Rev. Mol. Cell Biol..

[25] Wink D.A., Vodovotz Y., Laval J., Laval F., Dewhirst M.W., Mitchell J.B. (1998). The multifaceted roles of nitric oxide in cancer. Carcinogenesis.

[26] Fowley C., McHale A.P., McCaughan B., Fraix A., Sortino S., Callan J.F. (2015). Carbon quantum dot–NO photoreleaser nanohybrids for two-photon phototherapy of hypoxic tumors. Chem. Commun..

[27] Sodano F., Cavanagh R.J., Pearce A.K., Lazzarato L., Rolando B., Fraix A., Abelha T.F., Vasey C.E., Alexander C., Taresco V. (2020). Enhancing doxorubicin anticancer activity with a novel polymeric platform photoreleasing nitric oxide. Biomater. Sci..

[28] Fraix A., Conte C., Gazzano E., Riganti C., Quaglia F., Sortino S. (2020). Overcoming doxorubicin resistance with lipid-polymer hybrid nanoparticles photoreleasing nitric oxide. Mol. Pharm..

[29] Chegaev K., Fraix A., Gazzano E., Abd-Ellatef G.E.F., Blangetti M., Rolando B., Conoci S., Riganti C., Fruttero R., Gasco A. (2017). Light-regulated NO release as a novel strategy to overcome doxorubicin multidrug resistance. ACS Med. Chem. Lett..

[30] Fraix A., Parisi C., Failla M., Chegaev K., Spyrakis F., Lazzarato L., Fruttero R., Gasco A., Sortino S. (2020). NO release regulated by doxorubicin as green light-harvesting antenna. Chem. Commun..

[31] Parisi C., Moret F., Fraix A., Menilli L., Failla M., Sodano F., Conte C., Quaglia F., Reddi E., Sortino S. (2022). Doxorubicin-NO releaser molecular hybrid activatable by green light to overcome resistance in breast cancer cells. ACS Omega.

[32] Ghione S., Mabrouk N., Paul C., Bettaieb A., Plenchette S. (2020). Protein kinase inhibitor-based cancer therapies: Considering the potential of nitric oxide (NO) to improve cancer treatment. Biochem. Pharmacol..

[33] Laneri F., Graziano A.C.E., Seggio M., Fraix A., Malanga M., Beni S., Longobardi G., Conte C., Quaglia F., Sortino S. (2022). Enhancing the anticancer activity of sorafenib through its combination with a nitric oxide photodelivering β-cyclodextrin polymer. Molecules.

[34] Parisi C., Failla M., Fraix A., Rolando B., Gianquinto E., Spyrakis F., Gazzano E., Riganti C., Lazzarato L., Fruttero R. (2019). Fluorescent nitric oxide photodonors based on BODIPY and rhodamine antennae. Chem. Eur. J..

[35] Othman M., Bouchemal K., Couvreur P., Desmaële D., Morvan E., Pouget T., Gref R. (2011). A comprehensive study of the spontaneous formation of nanoassemblies in water by a “lock-and-key” interaction between two associative polymers. J. Colloid Interface Sci..

[36] Parisi C., Seggio M., Fraix A., Sortino S. (2020). A High-Performing Metal-Free Photoactivatable Nitric Oxide Donor with a Green Fluorescent Reporter. ChemPhotoChem.

[37] Montalti M., Credi A., Prodi L., Gandolfi M.T. (2006). Handbook of Photochemistry.

[38] Trotta F., Caldera F., Cavalli R., Mele A., Punta C., Melone L., Castiglione F., Rossi B., Ferro M., Crupi V. (2014). Synthesis and characterization of a hyper-branched water-soluble β-cyclodextrin polymer. Beilstein J. Org. Chem..

[39] Battistini E., Gianolio E., Gref R., Couvreur P., Fuzerova S., Othman M., Aime S., Badet B., Durand P. (2008). High-Relaxivity Magnetic Resonance Imaging (MRI) Contrast Agent Based on Supramolecular Assembly between a Gadolinium Chelate, a Modified Dextran, and Poly-β-Cyclodextrin. Chem. Eur. J..

[40] Daoud-Mahammed S., Couvreur P., Bouchemal K., Chéron M., Lebas G., Amiel C., Gref R. (2009). Cyclodextrin and Polysaccharide-Based Nanogels: Entrapment of Two Hydrophobic Molecules, Benzophenone and Tamoxifen. Biomacromolecules.

[41] Gidwani B., Vyas A. (2014). Synthesis, characterization and application of Epichlorohydrin-β-cyclodextrin polymer. Colloids Surf. B Biointerfaces.

[42] Swaminathan S., Garcia-Amorós J., Fraix A., Kandoth N., Sortino S., Raymo F.M. (2014). Photoresponsive polymer nanocarriers with a multifunctional cargo. Chem. Soc. Rev..

[43] Kandoth N., Kirejev V., Monti S., Gref R., Ericson M.B., Sortino S. (2014). Two-photon-fluorescence imaging and bimodal phototherapy of epidermal cancer cells with biocompatible self-assembled polymer nanoparticles. Biomacromolecules.

[44] Kirejev V., Kandoth N., Gref R., Ericson M.B., Sortino S. (2014). A polymer-based nanodevice for the photoregulated release of NO with two-photon fluorescence reporting in skin carcinoma cells. J. Mater. Chem. B.

[45] Fraix A., Kandoth N., Manet I., Cardile V., Graziano A.C.E., Gref R., Sortino S. (2013). An engineered nanoplatform for bimodal anticancer phototherapy with dual-color fluorescence detection of sensitizers. Chem. Commun..

[46] Deniz E., Kandoth N., Fraix A., Cardile V., Graziano A.C.E., Lo Furno D., Gref R., Raymo F.M., Sortino S. (2012). Photoinduced Fluorescence Activation and Nitric Oxide Release with Biocompatible Polymer Nanoparticles. Chem. Eur. J..

[47] Coriat R., Nicco C., Chéreau C., Mir O., Alexandre J., Ropert S., Weill B., Chaussade S., Goldwasser F., Batteux F. (2012). Sorafenib-induced hepatocellular carcinoma cell death depends on reactive oxygen species production in vitro and in vivo. Mol. Cancer Ther..

[48] Stępniak J., Krawczyk-Lipiec J., Lewiński A., Karbownik-Lewińska M. (2022). Sorafenib versus Lenvatinib Causes Stronger Oxidative Damage to Membrane Lipids in Noncancerous Tissues of the Thyroid, Liver, and Kidney: Effective Protection by Melatonin and Indole-3-Propionic Acid. Biomedicines.

[49] Goldstein S., Czapski G. (1995). The reaction of NO· with O^2−^ and HO^2−^: A pulse radiolysis study. Free Radical Biol. Med..

[50] Brinda B.J., Viganego F., Vo T., Dolan D., Fradley M.G. (2016). Anti-VEGF-Induced Hypertension: A Review of Pathophysiology and Treatment Options. Curr. Treat. Options Cardiovasc. Med..

[51] Kruzliak P., Kovacova G., Pechanova O. (2013). Therapeutic potential of nitric oxide donors in the prevention and treatment of angiogenesis-inhibitor-induced hypertension. Angiogenesis.

